# Leveraging Technology to Manage Chagas Disease by Tracking Domestic and Sylvatic Animal Hosts as Sentinels: A Systematic Review

**DOI:** 10.4269/ajtmh.19-0050

**Published:** 2019-09-23

**Authors:** Clemens Scott Kruse, David A. Guerra, Raena Gelillo-Smith, Amber Vargas, Laavanya Krishnan, Paula Stigler-Granados

**Affiliations:** Texas State University, San Marcos, Texas

## Abstract

Surveillance of Chagas in the United States show more is known about prevalence in animals and vectors than in humans. Leveraging health information technology (HIT) may augment surveillance efforts for Chagas disease (CD), given its ability to disseminate information through health information exchanges (HIE) and geographical information systems (GISs). This systematic review seeks to determine whether technological tracking of *Trypanosoma cruzi*–infected domestic and/or sylvatic animals as sentinels can serve as a potential surveillance resource to manage CD in the southern United States. A Boolean search string was used in PubMed and the Cumulative Index to Nursing and Allied Health Literature (CINAHL). Relevance of results was established and analysis of articles was performed by multiple reviewers. The overall Cohen statistic was 0.73, demonstrating moderate agreement among the study team. Four major themes were derived for this systematic review (*n* = 41): animals act as reservoir hosts to perpetuate CD, transmission to humans could be dependent on cohabitation proximity, variations in *T. cruzi* genotypes could lead to different clinical manifestations, and leveraging technology to track *T. cruzi* in domestic animals could reveal prevalent areas or “danger zones.” Overall, our systematic review identified that HIT can serve as a surveillance tool to manage CD. Health information technology can serve as a surveillance tool to manage CD. This can be accomplished by tracking domestic and/or sylvatic animals as sentinels within a GIS. Information can be disseminated through HIE for use by clinicians and public health officials to reach at-risk populations.

## INTRODUCTION

### Rationale.

Chagas disease (CD) or American trypanosomiasis is a zoonotic, vector-borne illness and a major public health threat.^1–4^ Caused by the protozoan parasite *Trypanosoma cruzi*, it is commonly found in endemic Latin American countries. The parasite is located in the feces of host animals, and it must be inoculated into the bite wound to enable transmission. Although the United States is considered to be a non-endemic country by the WHO, the parasite and the vector are commonly found in the southern United States, and local transmission has occurred.^1,3,5^ As a result of human migration, persons with CD can be found in non-endemic regions of the world and often left without resources to receive diagnosis and treatment for this serious disease.^2,5^ Chagas disease in humans is primarily transmitted via hematophagous insect vectors called triatomines (i.e., “kissing bugs”). The insect can transmit *T. cruzi* to the host by defecating onto bite wounds and/or mucosa where it can subsequently enter into the bloodstream. This can ultimately lead to distinct clinical manifestations of CD, which includes acute, chronic indeterminate (without symptoms), and possibly chronic determinant (with symptoms).^3–5^

Acute phase CD generally presents itself with either mild flu-like symptoms or can be asymptomatic, lasting approximately 8–10 weeks in duration.^1,3^ Many people are unaware they have been infected and fail to seek treatment during this phase. To complicate this phase even further, there are no commercial diagnostic tests in the United States that can be used to test for acute CD. In the United States, the only current testing recommendation to confirm acute CD is polymerase chain reaction testing that is presently only performed through the CDC laboratories. Many people will be completely unaware they have the disease for months or years because of the difficult diagnosis and the lack of symptoms. Whenever possible, it is important to identify and treat acute CD, which can help prevent the progression toward chronic CD.^6^

Persons living with chronic CD can often go years or decades without symptoms; this is called the chronic indeterminate phase. It is estimated that only about 30% of individuals with CD will go on to the determinant phase and develop debilitating cardiac and/or gastrointestinal complications.^1–3^ Treatment options are available; however, it is not recommended for adults older than 50 years or those persons presenting with CD complications. Failure to address CD in a timely manner can subsequently result in prolonged illness or death. There are several other modes of transmission of CD, including via contaminated blood supplies, organ transplantation with an infected organ, congenital transmission, and via food or drink. The United States screens blood donations from first-time blood donors; however, donors are generally not tested again because the assumption is that they are not living in an endemic CD country.^1^

To date, there are approximately 8–11 million people worldwide living with CD and as many as 10,000 of these individuals will die from the chronic complications mentioned.^1,2^ Still, the southern United States and the nation as a whole continue to disregard CD as a significant problem. This is despite known reports of autochthonous cases being documented in the southern United States. Since the start of blood donor screening initiatives, there have been 797 confirmed cases in 42 states.^5^ Nevertheless, many strongly believe that only 300,000 infected individuals reside in the United States and presume that CD is not concerning.^3,5,7^ It is important to realize that the previous statistic regarding the number of infected individuals was presented as an approximation of imported Hispanic cases and does not take into account other high-risk or misdiagnosed patient populations.

The approximation of individuals living in the United States who are infected with CD was calculated more than a decade ago and relied heavily on both CD in documented foreign-born immigrants and seropositive blood donations.^2^ This presents a severe underestimation of the true CD burden and fails to include in their calculations other at-risk populations such as undocumented immigrants, hunters, congenital cases, and idiopathic cardiomyopathy cases that may be undiagnosed CD.^4^ Furthermore, blood donations are voluntary and many higher risk populations such as immigrants are less likely to donate blood.^2^ Therefore, current methods used to track and monitor CD within the United States are undoubtedly inaccurate, leaving the true burden of CD ambiguous.^2,5^ The potential dangers of CD and the mere idea of underestimations in disease prevalence indicate a need for more reliable tracking methods, especially in the southern United States where there is confirmed autochthonous transmission.^5^ New solutions need to be endorsed to address the lack of available CD surveillance data. This is evident, as obtaining more information will help reveal potential at-risk populations within the southern United States. One possible method in overcoming the lack of data can be accomplished by implementing and using health information technology (HIT).

Health information technology should be used as an effective tool to connect public health officials and clinicians to meaningful health-care data via health information exchanges (HIEs).^8^ Access to shared data could help clinicians make definitive decisions in diagnosing CD infections that could ultimately lead to the detection of CD cases, as well as early intervention efforts for chronic phase progression. Furthermore, HIT in the form of HIEs could significantly improve population health management (PHM) of CD, enhancing the overall prognosis and clinical treatment outcomes. The CDC is developing initiatives specifically focused on HIEs to track disease occurrence. However, no such HIE initiative presently exists for tracking CD. One HIE medical informatics application that could be used involves implementing geographical information systems.^9^ However, a more prominent data source is needed to consistently surveil and manage CD, given that current sources are unreliable.

Infected domestic and/or sylvatic animal hosts with *T. cruzi* are a potential data source that can be tracked with HIEs.^10^ Presently, in the United States, there are 11 known species of triatomines that cumulatively have the potential to infect more than 100 different mammalian host species.^5^ Research suggests that understanding the relative importance of these animals as reservoir hosts could increase the overall understanding of CD infection across broad geographic bands. Published articles supplement this idea by confirming *T. cruzi* transmission cycles within both domestic and sylvatic hosts in Texas, which strongly views the United States as endemic for CD.^10–13^

This research is focused on the notion that it may be possible for animals to be used as HIT sentinels in tracking CD prevalence within the southern United States. In this context, animals would be used to reveal potential “danger zones or hotspots” for CD prevalence, allowing public health officials and clinicians the opportunity to identify areas where there could be potentially infected individuals. Awareness by clinicians of persons with a higher risk for CD living within these danger zones could help them to reconsider the likelihood or risk of *T. cruzi* infection, and, therefore, encourage laboratory testing and subsequent treatment plans.

## OBJECTIVES

The aim of this systematic review was to determine if HIT tracking of *T. cruzi* infected domestic and/or sylvatic animals as sentinels can serve as a potential surveillance resource to manage CD in the southern United States. This will be addressed by the following:

1. Investigating the correlation between CD prevalence and the appearance of domestic and/or sylvatic animal host species, as reported in the scientific literature
2. Assessing how HIT tracking of these seropositive animals as sentinels could reveal CD “danger zones” in the United States to help clinicians diagnose at-risk populations for autochthonous transmission

## METHODS

### Boolean search code and selection criteria.

This systematic review followed “a priori” design, formulating the Boolean search code, hypothesis, inclusion, and exclusion criteria before the study. Furthermore, the protocol adhered to guidelines provided by AMSTAR,^14^ the Preferred Reporting Items of Systematic Reviews and Meta-Analysis statement,^15^ and the Cochrane handbook.^16^ Several preliminary steps were followed to create a finalized Boolean search code that could produce an exhaustive search for this study. First, the study team consulted with a CD subject matter expert before data extraction. This was primarily performed to identify key terms and medical subject heading (MeSH) terms that covered the objectives of the systematic review. Recommendations were also applied to ascertain key terms that linked to *T. cruzi* animal hosts within the Boolean search code. The search string used was (“american trypanosomiasis” OR “trypanosoma cruzi” OR “t. cruzi” OR “chagas disease”) AND (“health information management” OR “surveillance” OR “trends” OR “economics” OR “education” OR “organization and administration” OR “sylvatic” AND “veterinary” OR “donations”) NOT (“primates” OR “bats”).

Next, the study team incorporated HIT MeSH terms within the Boolean search code. Note, the MeSH terms synonymously characterizes HIT as an entry term under the umbrella “medical informatics”; as such, qualifiers were used from this MeSH heading. The study team narrowed these HIT qualifiers further by requiring that the terms additionally be qualifiers listed specifically under both health information management and HIEs. These two MeSH headings fall directly under the HIT umbrella (also known as medical informatics) in accordance with the MeSH tree structure. This was completed to ensure the study team could effectively understand the ways in which HIT could track CD across the United States.

For instance, HIEs specifically involve the utilization of HIT to electronically disseminate clinical data across health-care settings. It also encompasses two other important MeSH headings: medical informatics applications and geographic information systems, a subheading of the latter. Medical informatics applications is the idea of using information systems to help communicate clinical data for diagnosis, thereby assisting in delivering patient care. Geographical information system is the utilization of computer systems to locate and map data collected. Recall that the study addresses the objective by assessing how HIT tracking of seropositive animals could reveal CD danger zones in the United States for the purpose of improved screening.

**Figure 1.**
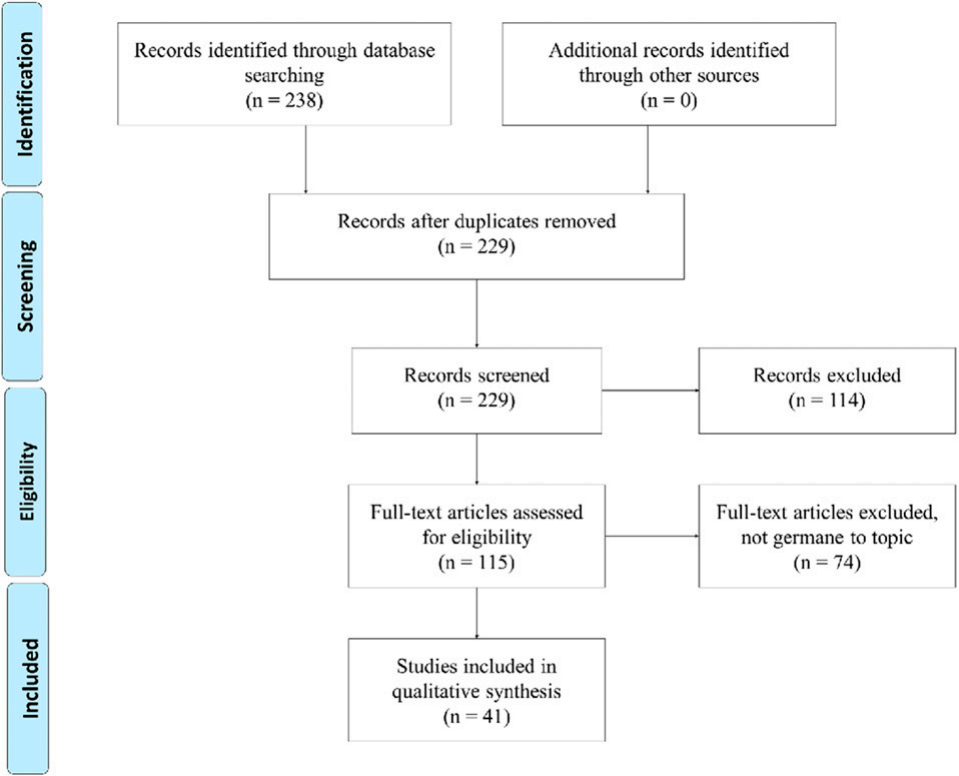
Preferred Reporting Items of Systematic Reviews and Meta-Analysis flow diagram illustrating the search process.

The study team concluded that HIEs through a medical informatic application such as a GIS could be used to assist with surveillance of CD within the United States. This could be accomplished by tracking *T. cruzi*–infected animals as sentinels to surveil CD prevalence. Health information exchanges incorporate themselves within the Boolean search code by encompassing all of the MeSH headings described to broaden the search and avoid exclusion of relevant articles. All of the terms used within the finalized Boolean search code are categorized within the Supplemental Appendix of this study. The finalized Boolean search code is illustrated in Figure 1.

Two electronic sources were used to perform the comprehensive literature search: PubMed and CINAHL. Both of these databases used the same Boolean search code to gather literature dating from January 1, 2007, to September 25, 2018. The study team accumulated literature starting from January 2007, to reflect the full historical timeline of CD prevalence within the United States. These included the following major political events that were not evident until January 2008: the implementation of voluntary testing for *T. cruzi* antibodies within U.S. blood donors and the Food and Drug Administration decision to enforce mandatory testing.^1^ Setting data parameters in such a manner allowed the study team to reflect any research that led both the United States and societies worldwide to investigate CD.

Other review eligibility criteria involved published articles that were in English only, peer reviewed, and available in full text. No limitations were provided regarding geographical location to deliver a robust search for the study. However, the study team did exclude articles that mentioned primates or bats for two different reasons. First, both primates and bats reflected newly emerging CD cases that were not extensively investigated to date. Second, CD cases in primates and bats were not species localized to the southern United States; thus, they were not pertinent to the scope of this study. Once these filters were applied, the data extraction process began.

### Data extraction.

The literature gathered from the Boolean search code was extracted via a group for analysis, consisting of three consensus meeting stages. The first consensus meeting was conducted after the Boolean search code was used to pull articles from both PubMed and CINAHL. Two reviewers were selected to read only the article abstract and collectively gauge whether or not to keep the journal for the study. Note that there were four reviewers in total that rotated during the data extraction process. Articles were selected if they primarily mentioned CD and/or animals as indicators of CD prevalence to widen the body of literature knowledge for a robust systematic review. Disagreements between reviewers within the study team at this stage were resolved by a third reviewer reading the abstract. Majority vote dictated consensus, if the third reviewer voted in favor of the literature abstract, then the article was kept and vice versa.

The second consensus meeting followed, this time analyzing the references of the articles chosen from the first consensus meeting. Similarly, the reference article abstracts were reviewed in the same fashion; however, the study team did not include any additional articles from these references for the final assessment review.

The third consensus meeting performed a full textual analysis of the articles and again articles were divided among two reviewers. Disagreements were not only resolved by a third member similarly to previous consensus meetings but also involved a consultation between the entire study team as deemed necessary. The elements presented within the final assessment allowed members to thoroughly gather emerging themes for a rich discussion within the systematic review. The overall risk bias of the data extraction was assessed by gauging interrater reliability among the four reviewers and was obtained by calculating Cohen’s kappa statistic.

## RESULTS

### Study selection, study characteristics, and results of individual studies.

The methodology literature search process for this systematic review is illustrated within Figure 1 and includes the consensus meeting results that occurred during the data extraction stage. The initial search gathered a total of 238 articles; 157 and 81 articles stemmed from PubMed and CINAHL, respectively. Application of eligibility criteria and duplicate removal minimized the group for analysis to 124 articles. As per the data extraction processes, these remaining articles were divided between rotating reviewers and subjected to three consensus meetings among the study team. This resulted in 41 articles being included within the study for subsequent review of emerging themes. The Cohen’s kappa statistic score, *k* = 0.73, reflected a moderate agreement among reviewers within the study team for this systematic review.

### Synthesis of results.

To synthesize results, the 41 articles selected were categorized based on the objective focuses specified prior. These foci sought to determine the potential for HIT to act as a surveillance resource for the management of CD within the southern United States and included the following: 1) investigating the correlation between CD prevalence in humans and the appearance of domestic or sylvatic hosts and 2) assessing how HIT tracking of these seropositive animals as sentinels could reveal CD danger zones for the purpose of helping clinicians diagnose at-risk human populations for autochthonous transmission. From here, two emerging themes were brought about from each focus, resulting in four themes in total: 1) domestic and/or sylvatic animals that act as reservoir hosts to perpetuate CD within the environment, 2) CD transmission to humans could be dependent on cohabitation proximity or contact between *T. cruzi* seropositive domestic and/or sylvatic species, 3) variations in *T. cruzi* genotypes could potentially lead to different clinical manifestations of the disease, and 4) using domestic and/or sylvatic animals as sentinels to provide a cost-effective source could reveal CD prevalent areas or “danger zones.”

The 41 articles were then divided among these themes to explain how HIT could be used as a potential surveillance resource through the use of animals as sentinels. Table 1 illustrates a summary of the group for analysis with explained summarizations and corresponding themes. Table 2 reflects an affinity matrix portraying a list of articles and frequency of occurrence for each theme.

**Table 1:** Summary of evidence from all articles analyzed depicting the key observations made and their corresponding theme into which we placed each observation

Author	Summary/relevance	Theme correlation
Curtis-Robles et al.^11^	Use citizen science program to spread awareness, track disease, and educate the public. Used morphological and molecular approaches to identify triatomines to learn about preferred habitats. Widespread occurrence throughout Texas.	2
Floridia-Yapur et al.^12^	Evaluated novel TcTASV protein family to detect active infection in dogs for faster detection.	3
Bennett et al.^17^	Increase surveillance activities to spread awareness and use data gathered by screening blood donors. No state included nonhuman data as part of public health surveillance, and, in 2017, CD became reportable in six states.	1
Vandermark et al.^18^	Confirmed first instance of wildlife *Trypanosoma cruzi* in Illinois, which suggests sylvatic life cycle in the region. Distribution of insects was tracked by strain to identify the spreading of the disease.	1, 2, 3
Wormington et al.^19^	Demonstrated the nocturnal risks of *T. cruzi* transmission by studying behavior and patterns of movement with various triatomine species.	1, 3
Aleman et al.^20^	Gathered *T. cruzi* data through rodent species in sylvatic and disturbed habitats. Rodents and insects gathered were at different seasons of the year and were accompanied by different species of triatomines.	1, 2
Arce-Fonseca et al.^21^	Called for an active surveillance program because of finding direct correlation of seropositivity between humans and dogs. Focusing on dogs may help to identify human prevalence of *T. cruzi* in infected areas.	1, 2
Gunter et al.^22^	Research suggests of sylvatic *T. cruzi* transmission cycle dating back to early 1900s and identified mammalian species that have been testing as *T. cruzi* positive in Texas. Additional research is needed to assess public health intervention and prevention efforts to prevent human infection.	1
Horney et al.^23^	Used the Community Assessment for Public Health Emergency Response (CASPER) to understand the risk factors associated with neglected tropical diseases (NTDs). CD was reported to regularly infect both humans and animals, and stray dogs within a community should be considered a risk for the disease.	1, 2
Curtis-Robles et al.^24^	*T. cruzi* was found to be prevalent in the raccoon population, with the majority being positive with TCIV strain when compared with other wild carnivores. Precautions need to be taken when individuals make contact with wildlife organs, especially hunters and wildlife professionals.	2, 3
Manne-Goehler et al.^25^	The findings of the study demonstrated a substantial burden of CD in the United States with many estimates not including undocumented immigrants. Awareness of the disease has increased in recent years, but further research needs to quantify prevalence to control CD.	4
Sanchez-Gonzalez et al.^26^	Various *T. cruzi* transmission pathways exists to humans, but the only prevention in Mexico has been based on heterogeneous blood donation screening, which has been instrumental in case detection. Cost savings were illustrated with screening efforts; however, compliance for *T. cruzi* requires greater governance.	4
Soriano‐Arandes et al.^13^	Presented a citizen science program as an effective way to generate data on the distribution and prevalence of triatomines. Program demonstrated public health benefit and community engagement while increasing surveillance efforts to control CD.	2, 4
Castillo-Neyra et al.^27^	Dogs are important reservoirs of *T. cruzi* and can reinitiate transmission after an area in sprayed with insecticide. Research suggests that dogs are useful in determining reemerging transmission and should be used to increase surveillance.	1, 2, 4
Curtis-Robles et al.^7^	Investigated CD by using a citizen science program to understand geographic distribution of kissing bugs and *T. cruzi* prevalence. Identified a high number of animals carrying the disease, which concluded that citizen science programs were an effective way to generate CD data for educating public and clinicians.	1
Perez et al.^28^	Explains the detrimental effects of reactivated CD could have on society in terms of prognosis. Eludes to the importance of active tracking of the disease at-risk population.	4
Valenasa-Barbosa et al.^29^	Gut contents of traitomines were analyzed to determine feeding sources for the insect. Study easily identified a sylvatic and domestic link to *T. cruzi* cycle. Led to another focus of oral transmission because of ingestion of *T. cruzi*.	1, 2
Esteve-Gassent et al.^30^	Emphasizes the vast distribution of CD across international borders. Focus was given on Texas in terms of risk factors due to socioeconomic status and living conditions that were consistent with disease prevalence in endemic countries.	2
Garcia et al.^31^	Provided general information of domestic and sylvatic cycles, underestimation of the disease, and at-risk population.	1, 2
Mejaa-Jaramillo et al.^32^	Attempted to identify *T. cruzi* genotypes in different communities by using laboratory testing. Demonstrated how human infection is linked to sociocultural conditions of humans living in close proximity to animals and insects. Specifically mentions the risk between domestic animals and sylvatic environment.	2
Soriano-Arandes et al.^33^	Investigated methods to reach out to CD-infected mothers and babies, mainly through congenital transmission. Demonstrated the need for surveillance, education, and pediatric awareness.	4
Tenney et al.^34^	Concluded that canine serosurveillance is a useful tool for public health risk assessment. Further stated that CD risk assessment can be identified through the use of shelter dogs and implies them to be wildlife reservoir hosts.	1, 4
Woodhall et al.^35^	Contains medical information regarding the treatment and symptoms of CD. Also outlines various diseases that physicians should focus on.	4
Carabarin-Lima et al.^36^	Most of the article expressed the importance of animals acting as sentinels and included information on infection prevalence.	1, 2, 4
Kessler et al.^37^	Lookback study used to analyze blood donations after the Food and Drug Administration approved a testing method for *T. cruzi*. Illustrates the potential CD prevalence in the United States.	1, 2
Kjos et al.^38^	Sought to understand CD host–vector–parasite relationship in the United States through the analysis of blood meal sources. Identifies domestic dogs as reservoir hosts that maintain peridomestic transmission cycles.	1, 2
Lee et al.^39^	Financial analysis of CD impact on societies worldwide. Reflect the financial cost of how CD can become a financial burden to a country.	4
Orozco et al.^40^	Four-year study over sylvatic transmission cycles via analyzing discrete typing units. Found that some animals can be more infectious than others and is possible for sylvatic and domestic cycles to separate.	1, 2
Thompson et al.^41^	Examines parasite zoonoses and wildlife in the context of the one health approach.	1, 2
Pineda et al.^42^	Study suggests that dogs are important in the peridomestic transmission cycle of *T. cruzi* as reservoirs and hosts.	1, 2, 4
Rosypal et al.^43^	Knowing that dogs are reservoirs for *T. cruzi* this quick/easy method of testing (ICD) can be administered to demonstrate more widespread testing.	4
Agapova et al.^44^	Demonstrated the cost-effectiveness of donor screening in the United States. Study promotes the use of donor screening and selective testing.	4
Schmunis and Yadon^45^	Follows the history of how CD spread from Latin American countries to become a global problem, mainly due to migration. Explains how the disease made its route to various countries and calls for government policies to detect/treat acute and chronic cases.	4
Castro^46^	Discussed the importance of protecting the blood supply from CD and whether universal screening efforts would be cost-effective for preventing infection. Continued to discuss blood screening for disease based on country.	4
Galuppo et al.^47^	Research revealed how different types of *T. cruzi* strains could be distributed and how it can effect reservoir hosts.	1, 2
Cardinal et al.^48^	Assessed an area with high *T. cruzi* infection to understand the distribution of lineages. Domestic dogs and cats were found to be infected with the virus, which supports both acting as reservoir hosts of *T. cruzi*.	1, 2
Castillo-Riquelme et al.^49^	Determined whether cost-effectiveness of blood screening policies were beneficial to increasing disability-adjusted life years and lowering costs. Results suggest greater tracking efforts and governance with CD policies.	4
Piron et al.^50^	Introduced a blood-screening program for CD for at-risk donors, but discovered seropositive individuals who were not residing in endemic countries. Importance of screening efforts were realized as *T. cruzi* screening is not performed routinely for all donations.	4
Roque et al.^51^	Compared three different environmental areas for *T. cruzi* transmission cycles and found that land use was a determinant factor in virus prevalence. The most common feature found in the study was the infection of dogs with the virus and stresses importance of using domestic animals as sentinels in the identification of *T. cruzi* transmission hot spots.	1, 4
Cardinal et al.^52^	Relative impact that *T. cruzi* had on houses was determined to be very high where control actions were not taken. Most of the infected animals qualified as autochthonous cases and domestic infestation led to a sharp increase in the likelihood of human infection.	1, 2, 4
Hanford et al.^53^	Stressed the lack of awareness of CD in Texas even though the disease is endemic and an emerging disease. Those infected with *T. cruzi* placed increased burden onto the health system and more appropriate measures should be taken when addressing health-care services and reportable diseases.	1, 4

CD = Chagas disease.

**Table 2:** Additional analysis

Main themes	Reference number	Frequency
1. Domestic and/or sylvatic animals that act as reservoir hosts to perpetuate CD within the environment.	7, 17, 19–23, 29, 31, 34, 37, 38, 40, 41, 47, 48, 51, and 53	18
2. CD transmission to humans could be dependent on cohabitation proximity or contact between *Trypanosoma cruzi* seropositive domestic and/or sylvatic species.	11, 13, 20, 21, 23, 24, 29–32, 37, 38, 40, 41, 47, and 48	16
3. Variations in *T. cruzi* genotypes could potentially lead to different clinical manifestations of the disease.	12, 19, and 24	3
4. Using domestic and/or sylvatic animals as sentinels to provide a cost-effective source could reveal CD prevalent areas or “danger zones.”	13, 25, 26, 28, 33–35, 39, 43, 44, 45, 46, 49–51, and 53	16
Total^α^	18, 27, 36, 42, and 52	5

CD = Chagas disease.

The specific articles associated with themes and their frequency of occurrence. Total^α^ refers to research articles that were included in three or more themes.

## DISCUSSION

### Summary of evidence.

The 41 articles used for this study generated four major themes (Table 2). These themes collectively revealed the underlying ability for HIT to surveil and manage CD within the southern United States. The aims mentioned within the systematic review objective statement each had two corresponding themes. These themes are described in detail in the following paragraphs.

#### Aim 1: Investigating the correlation between CD prevalence and the appearance of domestic and/or sylvatic animal host species.

The first theme associated with this aim involves both domestic and sylvatic animals acting as reservoir hosts to perpetuate CD within the environment. Several studies in both the United States and endemic countries have come to this conclusion. One article from Colombia suggested that domesticated dogs were susceptible to CD infection, despite frequent *T. cruzi* occurrences in sylvatic cycles.^41^ This is especially true if dogs frequently stay outdoors as proven by a citizen science program which found that 69–82% of kissing bugs collected from houses and dog kennels in central Texas were infected.^7^ In fact, dogs and wild or sylvatic hosts were suggested to act as reservoirs for *T. cruzi* according to a study in Sonora, Mexico.^21,42,47,48^

Another study in Brazil supplemented this first theme by adding two other domestic animals other than dogs that can perpetuate *T. cruzi* in the environment: chickens and goats. The study suggested a crucial relationship that links *T. cruzi* perpetuation from domestic to sylvatic cycles, allowing CD continuation within the environment.^29^ This same notion was consistent with other studies in the southern United States and it was found that various animals sustained *T. cruzi* transmission within the sylvatic cycle.^22^ These animals included, but are not limited to, the following: armadillos, raccoons, opossums, bobcats, coyotes, and foxes.^20,24^ One study revealed via a blood analysis that *Trypanosoma gerstaeckeri*, one of the most common species of kissing bugs in the United States, primarily feeds on nocturnal sylvatic species.^19^ It was also ill advised to have domestic animals outdoors in areas where this kissing bug resides because of the possible occurrence of *T. cruzi* transmission and CD susceptibility. It should be noted that *T. gerstaeckeri* is only one of 11 known species of kissing bugs that carry *T. cruzi* within the United States. Furthermore, *T. cruzi* has been known to infect more than 100 different types of mammalian hosts, thereby supporting two ideas: 1) that the parasite is actively being transmitted within the environment and that 2) the southern United States has already reached endemic status.^20,38^ It is possible that many mammalian hosts are infected via oral transmission by either digesting an infected animal or other *T. cruzi*–positive insects.^42,53^ One article provides evidence that supports this notion through several experimental reports that have documented the transmission of *T. cruzi* to opossums via ingesting infected mice.^51^

A second theme centered around the notion that CD transmission to humans could be dependent on cohabitation proximity or contact between *T. cruzi*–seropositive domestic and/or sylvatic species.^32,37,40^ Articles from endemic countries reflected this theme on numerous occasions. First, two studies from Mexico claimed that homes having dogs increased the risk of CD transmission to humans 5-fold.^21^ Another study even asserted a direct association between CD infections in canines and an increased risk of *T. cruzi* transmission to humans in the United States.^34^ This is because the seroprevalence of dogs (ranging from 17.5% to 21%) was linked to CD outbreaks in humans within two Mexican regions: including one where CD was not previously considered endemic.^34,36^ Another study in Brazos County, Texas, indicated that the presence of infected dogs, including strays, in or around the house were risk factors for CD to humans; chickens were also cited.^23^ Sylvatic animal hosts in the United States also posed a risk toward humans because they tend to establish populations near domestic settings; as such, species of kissing bugs continue to cause a spillover of *T. cruzi* transmission.^11^

The presence of these animals were not the only worrisome factor entailed within this theme. Direct contact with blood and tissue of wild animals infected with *T. cruzi* can potentially pose a health risk to hunters and wildlife professionals. In the southern United States, there are 24 wildlife species that are considered hosts of *T. cruzi*, and, in Texas, six of the 24 species are hunted year-round. This only strengthens the increased risks and dangers associated with CD transmission from animals to humans.^31^ These combinations of articles support the idea that there is a strong correlation between the appearance of animal hosts and the widespread occurrence of *T. cruzi* infection.

#### Objective Aim 2: Assessing how HIT tracking of these seropositive animals as sentinels could reveal CD “danger zones” in the United States for the purpose of helping clinicians diagnose at-risk populations for autochthonous transmission.

The first theme that corresponded with the second objective aim found that variations in *T. cruzi* genotypes could potentially lead to different clinical manifestations of the disease. Two studies in the United States demonstrated this idea by contrasting two *T. cruzi* strains, TcIV and TcI. The first study by Curtis-Robles mentioned that the southern United States has both strains present in kissing bugs and sylvatic animals, sometimes even mixed infection with both strains occurs within animals.^24^ Furthermore, the study specified that human cases were typically typed as having TcI strains.^18,24^ Another article revealed that TcIV-infected mice and rats experienced less mortality and morbidity from this strain.^18^ Understanding these two findings increases the possibility that TcI is more likely to cause the expression of CD infection in the form of cardiac disease in host species and possibly humans versus TcIV.^12^ Although these concepts are novel findings, they demonstrate the importance of understanding the role in genetic variation in managing CD.

The last theme for this aim entailed the use of domestic and sylvatic animals as cost-effective sentinels; this was for the purpose of revealing CD-prevalent areas (dubbed “danger zones”). There were several articles that concluded this theme and dogs were frequently cited as probable animal sentinels for tracking CD occurrences.^34^ For example, one reference asserted that *T. cruzi*–infected dog populations allowed for the identification of houses and clusters that were susceptible for CD transmission.^52^ Moreover, seropositive dogs were capable of forecasting the reemergence of *T. cruzi* populations as they were spatially linked to zones of CD infection.^13,27,28^ The significance of *T. cruzi*–infected canines in homes was also noted when they were found to increase the risk of CD transmission to humans by five times the norm.^52^

Clearly, there is explicit evidence of this literary theme. However, there were additional key implications, specifically in regard to the overall importance of surveillance efforts that were available in the group for analysis. This was conveyed in two ways, which all lead to the conclusion that there is a benefit in using animals as sentinels in this manner: 1) its ability to act as a surveillance effort that can potentially lower CD morbidity and mortality and 2) its ability to act as a more reliable lower cost surveillance method.

As mentioned prior, the first aspect portrayed by the literature at hand demonstrates that implementation of a surveillance program can prevent lifelong morbidities and overall mortality.^13^ It is important to understand that the United States unfortunately does not have an established national CD surveillance program.^17^ As such, CD surveillance efforts in the United States are severely lacking, causing many at-risk persons to remain unidentified.^25^ This can ultimately lead to significant morbidity and mortality that would have ultimately been prevented among at-risk populations such as *T. cruzi*–seropositive pregnant mothers had there been more stringent surveillance efforts.^13,33^

Interestingly, CD surveillance in one study was recommended for states that had a large at-risk populations that were 1) exposed to endemic countries or 2) infected mammalian reservoirs locally.^25^ Despite the last provision, the United States surprisingly discontinued collecting nonhuman data, deciding not to track animals as sentinels as it was deemed unnecessary. It is known blood donations that primarily provide relatively sparse surveillance data leaving gaps in information; nonetheless, this is the primary mode in which data on CD in the United States are gathered. Together, these ideas cumulatively encourage surveilling domestic and sylvatic animals as sentinels for the purpose of serving as PHM program that can deliver quality of care for at-risk populations.

Finally, cost-effectiveness is demonstrated through literary examples that portray the detriment of not surveilling animals as sentinels. Although various countries were assessed via a Markov simulation, it was noted that annual U.S. Chagas disease health-care costs equated to a mean of US$118,178,896 (range US$5,567,227–596,130,169).^39^ This amount was alarmingly high and rivals the spending seen in Brazil, which is the country with the highest CD burden in the world. Daily adjusted light years in the United States specifically also averaged to 27,590 (range 1,798–99,210), which was greater than most infected countries.^39^ Another study in Mexico added to the idea of cost-effectiveness through laboratory testing. It was found that costs for detected and undetected cases amounted to US$3.2 million; health-care and labor costs comprised 62.9% of this code.^26^ This information alone demonstrates plausibility of using animals to alleviate this sector as an outreach program and implies the importance of surveillance over the long term. Finally, the importance of control efforts in lowering costs was conveyed; using animals as sentinels can lead to control efforts via the key surveillance aspects discussed prior.^49^

## CONCLUSION

The goal of this systematic review was to determine whether HIT tracking of domestic and/or sylvatic animals as sentinels could serve as a potential surveillance resource to manage CD occurring within the southern United States. Based on the group for analysis, the study team proved their hypothesis concluding that HIT can be a tool to encourage the prevention and management of CD. This can be accomplished by using *T. cruzi*–infected domestic and/or sylvatic animals as sentinels. Understanding the two aims that bridged the relationship between CD infection in humans and animals should be a focal point when providing governance or policies. There were a total of four major themes that were derived from this study and each two themes paired to the described objective statement aims. We picked these four themes for readability purposes for the general audience. These themes all had great significance to the overall research question, but one theme truly bridged the management of CD with HIT: the benefit of using domestic and sylvatic species as beacons to highlight areas reflecting high *T. cruzi* transmission areas (also known as “danger zones” or “hotspots”) to humans. This was the most pertinent and critical finding for which the study team unanimously agreedon. This research couples nicely with the other work with its leverage of HIT. As such, one limitation within this study was the availability of articles explicitly mentioning HIT. There were a number of articles that heavily supported this theme as depicted throughout this systematic review.

In this context, HIT has the ability to expose *T. cruzi* “danger zones” or “hotspots” to instill awareness of CD within the southern United States. This can be achieved by tracking the overall frequency of domestic and/or sylvatic animal hosts as sentinels to provide the HIT data necessary to overlay and map *T. cruzi* prevalence. This could be accomplished via a medical informatics application such as a GIS, which can use data to spatially distribute and map *T. cruzi* across the southern United States. This allows HIT to be used as a surveillance resource in two ways. First, establishing this GIS and making concrete data available via an HIE could allow U.S. clinicians to diagnose potential CD cases swiftly. With this tool, clinicians can visually see local *T. cruzi* “danger zones”: should a patient live or come across to these “danger zones,” a clinician may be alerted to consider CD as a possible diagnosis. It would also promote preemptive, selective testing which may be a more cost-effective initiative for laboratories and societies’ worldwide, including the United States.^46^ Together, this satisfies aspects within the iron triangle by minimizing costliness of care all while increasing access to care and quality. It also encourages collaboration among stakeholders such as specialists, primary care providers, veterinarians, and public health officials who can use these data to treat and implement initiatives for those infected.

From a public health perspective, seeing HIT being used in this manner could potentially promote its adoption. This 1) deters diagnosis postponements of CD and 2) integrates usage of PHM, given that it helps identify at-risk human populations for *T. cruzi* transmission.^52^ By reaching out to these populations, the United States can achieve the Triple Aim approach by simultaneously reaching PHM goals and boosting the overall patient experience through positive CD prognoses. It is evident that HIT has policy implications by improving aspects of the Iron Triangle and achieving goals from the Triple Aim. Evidence of this work points to three recommends: 1) the United States should develop an online reporting tool to upload confirmed positive reports of animals as vectors, 2) use citizen science and collaborative groups to assist with surveillance for the southern U.S. states where reporting is not mandatory, and 3) support improved communication for veterinarians to discuss human health risks with their clients of pets who test positive for CD. These could truly help areas of suspected endemicity such as the southern United States changing the way in which the nation surveils CD to date.

Unfortunately, as of December 2017, only Arizona, Arkansas, Louisiana, Mississippi, Tennessee, and Texas conduct surveillance for Chagas disease and have made it a reportable condition. Chagas disease–positive animals are presently not being reported to any state agencies, however, many state veterinary laboratories are conducting the testing. The lack of tracking of positive animals in all states and positive vectors in those southern U.S. states where it is not reportable would make them difficult to track. However, our aforementioned recommendations would address this issue. Promotion of the tool use would be challenging and need to be part of an overall educational campaign on the disease.

## Supplemental appendix

Supplemental materials
