# Supplementary material for: Leveraging Technology to Manage Chagas Disease by Tracking Domestic and Sylvatic Animal Hosts as Sentinels: A Systematic Review
Source: Am J Trop Med Hyg. 2019 Sep 23;101(5):1126–34. doi: 10.4269/ajtmh.19-0050 (PMC6838565; doi:10.4269/ajtmh.19-0050)
Supplement: Supplementary file 1 [file tpmd190050.SD1.pdf]

Supplemental Appendix

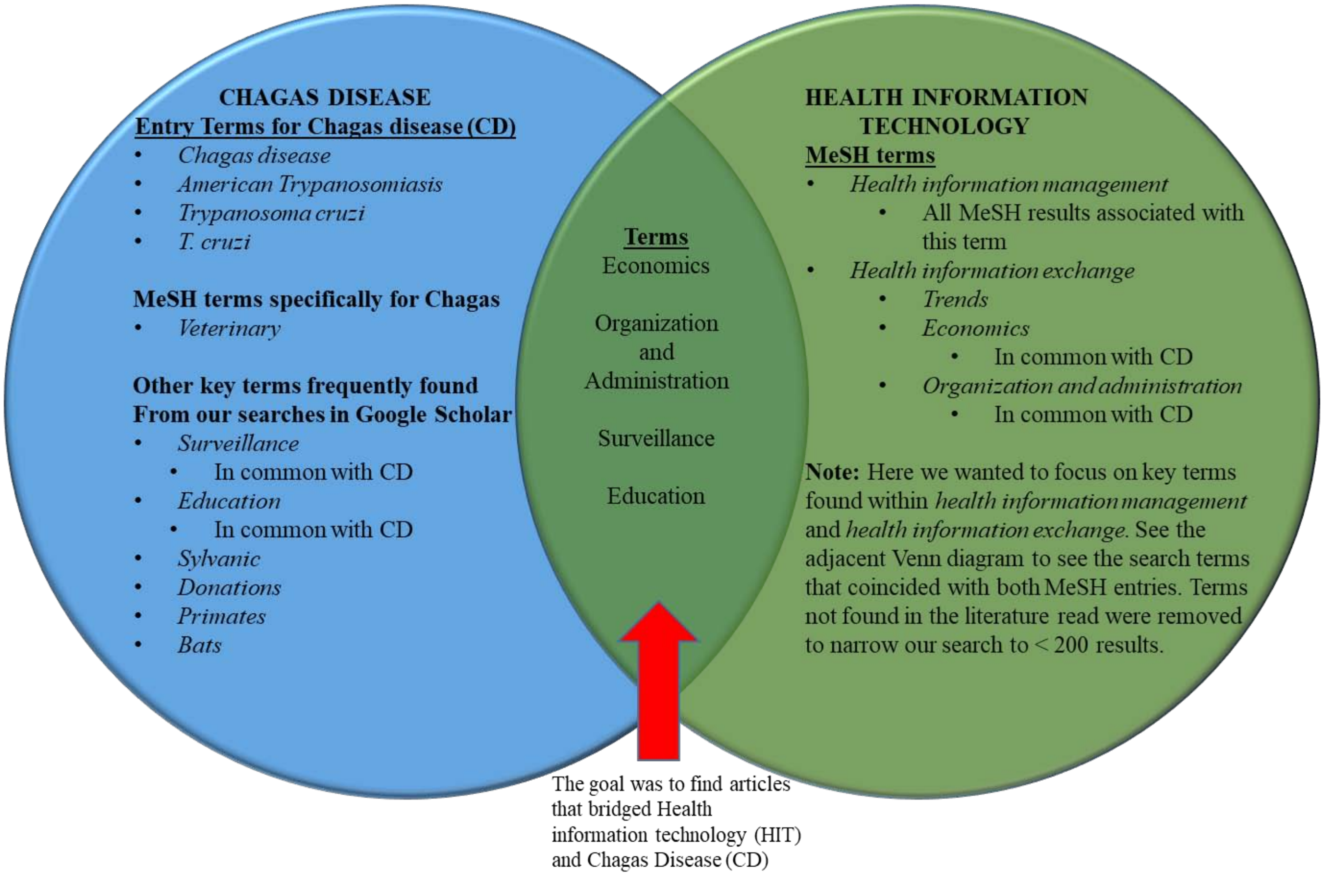

Venn Diagram that demonstrates a breakdown of the MeSH terms in detail based on the search criterion. The intersection defines the key terms used in our search: the relationship between Chagas Disease and Health Information Technology.

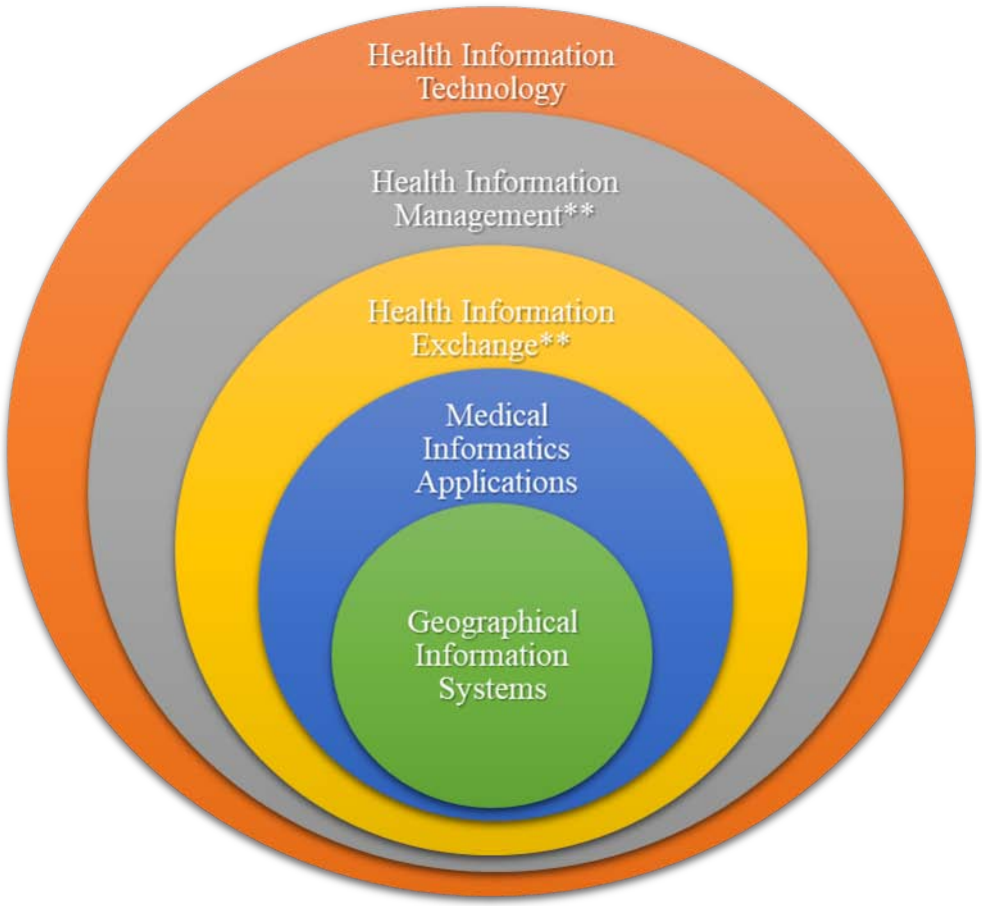

Concentric circles that demonstrate how each MeSH term nests into one another.
